# Evaluating the Provision of Health Services and Barriers to Treatment for Chronic Diseases among Syrian Refugees in Turkey: A Review of Literature and Stakeholder Interviews

**DOI:** 10.3390/ijerph16152660

**Published:** 2019-07-25

**Authors:** Jude Alawa, Parmida Zarei, Kaveh Khoshnood

**Affiliations:** 1 Yale University, New Haven, CT 06520, USA; 2 Department of Chronic Disease Epidemiology, Yale School of Public Health, New Haven, CT 06510, USA; 3 Department of Epidemiology of Microbial Diseases, Yale School of Public Health, New Haven, CT 06510, USA

**Keywords:** Global Health, Migrant Health, Refugee Health, Syrian Refugees, Turkey, Chronic Disease, Healthcare Access, Barriers to Care

## Abstract

Background: While Turkey hosts the largest number of Syrian refugees, the provision of health services for chronic disease among Syrian refugees in Turkey has been inadequate and understudied. This paper explores Turkish healthcare policies surrounding Syrian refugees’ access to health services for chronic diseases. Methods: We conducted a literature review and supplementary stakeholder interviews to evaluate the provision of chronic health services and the most common barriers to healthcare access among Syrian refugees in Turkey. Results: Though access to treatment for displaced Syrians has improved throughout the past five years, five primary barriers persist: registration procedure regulations, navigation of a new health system, language barriers, fear of adverse treatment, and cost. Conclusions: To drive improvements in healthcare for chronic diseases among Syrian refugees in Turkey, we recommend making registration procedures more accessible, developing more healthcare options in patients’ native language, increasing human resources, and advocating for more research surrounding chronic health conditions among refugees.

## 1. Introduction

The conflict in Syria has produced over 5.3 million registered Syrian refugees, and Turkey currently hosts over 60 percent of this population [1]. Migration, as a result of fleeing persecution and seeking asylum in host countries, is associated with adverse health outcomes [2]. Since refugees are often forced into shantytowns and slums, they face living conditions that perpetuate a lack of physical activity, poor nutrition, and tobacco consumption—well-established risk factors for chronic disease [3]. There is a high burden of chronic disease among urban refugees in the Middle East, ranging from 9% to 50% depending on the refugees’ country of origin. Specifically, hypertension, musculoskeletal disease, diabetes, and chronic respiratory disease are some of the most prevalent chronic diseases that contribute to morbidity and mortality among this population [4]. In a 2019 survey of 10,019 Syrian refugees living in Turkey, 15.2% of respondents reported having a chronic disease, with hypertension, psychiatric disorders, and diabetes reflecting the most common chronic diseases reported [5]. Further, a health status survey by the World Health Organization reports that over 50 percent of Syrian refugees in Turkey are at high risk—defined as having 3–5 risk factors—for developing a chronic disease [6]. Although Turkey hosts the greatest proportion of Syrian refugees, there has been limited research bringing to light the high risk and burden of chronic disease, primary health care access, and the provision of health services for this particular demographic. The prevalence of chronic disease among Syrian refugees living in Turkey combined with the dearth of research in this area warrants the urgent need to further understand and address the health needs of this population. 

Since chronic conditions, such as diabetes and cardiovascular disease, require sustained care coordination and treatment, migrant populations may experience disruptions in treatment, as they resettle in Turkey and navigate access to a new health system. Lapses in treatment and delayed care may lead to poorly managed health conditions, potential complications, and several comorbidities. It is unclear whether the Turkish healthcare system is sufficiently equipped to address the chronic disease burden of its refugee population, especially in tandem with the high burden of chronic disease within its host population [7]. As such, there is a need to ensure that the burden of chronic disease among the Syrian refugee population in Turkey is adequately addressed through sustainable and equitable access to healthcare services [8,9]. Traditionally, host health systems and research efforts have prioritized emergency needs and infectious diseases to address immediate health needs spurred as a result of travel and poor health conditions in the country of origin and to contain the spread of disease [10,11]. Non-communicable diseases have received less traction, perhaps because of the challenges of providing continuous care to a large population and the less urgent nature by which these conditions typically transpire. A recent systematic review reveals that there are currently no data addressing access to chronic disease services for Syrian refugees in Turkey, highlighting a need for research in this area to illuminate refugee health needs and gaps in care [12]. 

The high prevalence of non-communicable diseases, disruptions in care following migration, and the limited research in this area within the Turkish context present a prime opportunity to not only make strides toward preventing, detecting, and treating chronic diseases among Syrian refugees, but also to strive toward creating a more accessible healthcare system in host countries including Turkey [13,14]. In addition to Turkey’s over 3.5 million registered Syrian refugees, Turkey also hosts estimates of hundreds of thousands to over a million unregistered Syrian refugees who have no access to public health care. Moreover, from an economic perspective, the burden is immense, with Turkey reportedly having already spent over $30 billion on services for Syrian refugees, including $10 billion on health services alone [15]. Given the rapid influx of Syrian refugees into Turkey and the challenges of providing healthcare for this large, vulnerable population, an investigation into Turkey’s changing asylum and healthcare policies and their impact on Syrian refugees in Turkey is necessary. This paper examines Turkish healthcare policies for Syrian refugees and how they have affected Syrian refugees’ access to health services for chronic diseases and aims to bring more attention to the risk and burden of non-communicable diseases among this growing population in Turkey. 

## 2. Materials and Methods

A literature review and supplementary interviews with stakeholders to the Syrian refugee crisis were conducted. The literature review focused on identifying peer-reviewed publications in English that pertain to Turkish healthcare policies, migrant health in Turkey, health outcomes for displaced populations in Turkey, chronic diseases among Syrian refugees in Turkey, and the provision of refugee health services in Turkey. We also searched for non-peer reviewed sources from entities including the Turkish Ministry of Health and the United Nations. Our literature review of government and non-government publications provided insight into specific gaps and questions surrounding access to healthcare for chronic disease among Syrian refugees in Turkey that were then explored through supplementary interviews.

Sixty-one stakeholders within Turkey were interviewed by convenience sampling using a semi-structured interview format. Criteria for selection included direct involvement in the provision of services for those displaced or involvement with an organization directly working on the matter of displacement in Turkey. Stakeholders included nine personnel from think tanks, three from academic institutions, 23 from humanitarian non-profit and non-governmental organizations, 11 from international agencies, and 15 from government entities. All interviewees and information collected were anonymous. In a protracted humanitarian crisis such as that in Turkey, the delivery of services, specifically for healthcare, tends to differ and become more nuanced as compared to written government laws and policies. As such, the purpose of the interviews was to identify the gaps and nuances in the literature surrounding healthcare access in Turkey.

Together, our literature review, interviews, and acquired unpublished materials from stakeholders provide meaningful information to elucidate the current situation of access to health services among Syrian refugees in Turkey, especially as they pertain to the provision of health services for chronic diseases. We have presented our results as a detailed breakdown of the Turkish health system and healthcare access, specifically for chronic disease, among Syrian refugees. This study was approved by the Yale University Institutional Review Board. 

## 3. Results

### 3.1. The Turkish Healthcare System

In 2013, the Turkish healthcare system began a series of health system reforms under the Turkish Health Transformation Programme (HTP). The HTP was primarily aimed at improving healthcare access, delivery, and quality, and addressing the rising prevalence of chronic disease among the aging population. Under the HTP, Turkey has moved toward universal health coverage and public financing through the development of a social security system [16].

The Turkish Ministry of Health operates and administers all health facilities within the Turkish healthcare system by way of provincial health directorates. In each of the country’s governorates, the Ministry of Health ensures that health providers, including both public and private sector contributors, act in compliance with Turkish policies. Since 2008, however, the Ministry of Labour and Social Security has been tasked with financing public health insurance under an entity called the Social Security Institution (SSI). SSI is considered a single payer, in which it uses contributory health insurance and government funds to strategically purchase healthcare from providers. Similarly, the establishment of the SSI has led to a General Health Insurance Scheme (GHIS), which consolidates five different health insurance schemes in Turkey into one general scheme that supposedly guarantees access to expanded provider benefits [17,18]. Beyond public insurance, individuals can elect to purchase private health insurance for additional benefits and services. 

Under the GHIS, all registered individuals, regardless of their given identity as a migrant or citizen, are guaranteed universal access to healthcare services [19]. More specifically, the GHIS is intended to cover “all citizens who were covered by previous social security laws and their dependents, foreign residents who do not have social security coverage in their home countries, individuals benefiting from unemployment insurance, and those under the protection of the Foreigners International Protection Law [20]”. Thus, any migrant who registers in a Turkish governate and receives an identity card confirming their residency status within Turkey is eligible for access to health services. However, under the Foreigners International Protection Law, unregistered migrants are not guaranteed access to care. 

Each year, the Ministry of Health publishes a report called the Health Implementation Guide that details the rules, regulations, and services covered under GHIS, including the prices of particular healthcare services. Most treatments, including emergency services, fall within the public health insurance scheme, where individuals can utilize free public hospitals to access care. The provision of these free health services is based on the Social Insurance and Universal Health Insurance Law of the Turkish Constitution [21]. If any service is not completely covered per the Health Implementation Guide, the cost of the service or its partial coverage will be detailed in the Guide.

For the host population, HTP implementation has significantly improved healthcare in Turkey, including reductions in out-of-pocket spending for health services, increases in human resources, improvements in patient satisfaction, and greater health insurance coverage, especially among the lowest income groups. [16,17,22]. Turkey is one of several countries that have been identified as experiencing some of the largest gains in healthcare access and quality between 1990 and 2015 [23].

The Ministry of Health has identified disease prevention and health promotion as priorities during Turkey’s health reform, and the HTP has directed much of its efforts to strengthening primary health care. A fundamental change in primary care in Turkey under the HTP has been the introduction of the Family Medicine model [24]. Key features of the Family Medicine model include assignment of a family medicine doctor to each individual, re-training of primary care physicians, and increased preventive care activities [25]. These reform efforts have had significant implications on the state of primary health care in Turkey, including greater funding for primary health care services, increased physical and human resources and capacity, and expanded preventive care services. Most notably, cost-sharing for primary health care services was abolished under the HTP, and these services are now provided free of charge for Turkish citizens [17,25]. Despite these reforms, primary care in Turkey is still not sufficiently equipped to confront the high chronic disease burden of its population, especially given the additional strain placed on the health system, as a result of the influx of Syrian refugees [17,22].

### 3.2. Syrian Refugees’ Access to Healthcare in Turkey

Legal status is a fundamental determinant in defining how migrants can interact with the Turkish healthcare system. Turkey has historically maintained a number of legal limitations relating to asylum seekers. Though it has been noted that some Syrian migrants in Turkey have been granted citizenship by naturalization, Syrian refugees’ right to exist and their right to services within Turkey is guaranteed under the Temporary Protection Regulation (TPR) within the Turkish Constitution. TPR is given to Syrian asylum-seekers in Turkey who have officially registered with the United Nations Higher Commission on Refugees (UNHCR) or an associated government entity. TPR has been in effect since October 2014 and has dictated that temporary protection identity cards be given to all registered Syrians. These identity cards grant Syrian migrants access to legal rights and social services, such as healthcare, education, and most recently, work permits. However, ensuring timely and accessible registration procedures, while essential to promoting refugees’ healthcare access, is not sufficient to address the chronic health concerns of refugees. For example, the distribution of identity cards does not definitively guarantee that vulnerable populations will be able to access health and social services. Barriers and challenges remain, especially in the provision of health services for a large population of Syrian migrants. Consequently, a multi-pronged approach to improving healthcare access and quality is needed.

Before the provision of TPR for Syrian migrants, Syrians had no official legal status or rights within Turkey, including access to healthcare [26]. When the rapid influx of Syrians into Turkey began, the first tent settlements were established near the Syrian border in Yayladağı (Hatay) and were managed by the Disaster and Emergency Management Presidency in Turkey (AFAD). AFAD was tasked with the regulation and provision of all services related to Syrian migrants. Since the institutional framework of AFAD was set-up to respond to crisis situations, AFAD’s Regulation on the Center for Disaster and Emergency Management was used as the legal basis for the provision of healthcare services to Syrian refugees [27,28].

As the number of Syrian asylum seekers increased, Turkish policymakers responded in 2013 by increasing access to health services for migrants. However, because no specific regulations were set in place for Syrian refugees, the implementation of such policies were fragmented and ineffective, making access to medical treatment limited. With the establishment of the Migration Health Services Head Office as a part of the Ministry of Health to coordinate health services for migrants, policymakers developed a new law in November 2015 entitled, “The Fundamentals of Health Services to be delivered to those under Temporary Protection”. This law tasked the Ministry of Health with the responsibility of providing Syrian migrants under Temporary Protection with all primary health services, including preventative care, immunization, environmental health services, women’s and reproductive health services, child health services, and vaccinations [29]. Under Temporary Protection, Syrians residing in Turkey were provided with free access to healthcare services but only if they were registered with the UNHCR or an associated government institution, as a temporarily protected person. Without registration, Syrian migrants would not be able to access any free health services, except those stipulated above as a part of emergency healthcare. Additionally, though most Syrians living in camps are required to be registered, all government managed camps in Turkey provide free access to primary health care facilities staffed by personnel from AFAD. Patients can also be referred from the camps to public hospitals depending on the complexity of their case [30]. 

The provision of chronic health services for Syrian refugees is the responsibility of AFAD. With the establishment of the Family Medicine model under the HTP came the development of family health centers that served as the main provider of primary health services for Syrian refugees [31]. In 2015, additional health facilities called Migrant Health Centers were established to provide further support for the Syrian refugee population, particularly in areas densely populated with Syrian refugees. The main goal of the Migrant Health Centers was to address the language barriers and stigma frequently encountered at Turkish community health centers. Presently, the Migrant Health Centers serve as the main pillar for the primary care services available to Syrian refugees. 

These centers, part of a larger effort between the Ministry of Health and the European Union and financed by a grant from the European Union, are aimed at offering widespread and effective primary health care to migrants, specifically Syrians under TPR. Each Migrant Health Center provides primary health care services and also employs Syrian doctors or interpreters to facilitate the provision of health services. Language barriers have been cited as a significant barrier to care among Syrian refugees, so the presence of interpreters has been noted to significantly increase willingness to seek health services. Not only does this provide an avenue for Syrian migrants to be better equipped to seek health services, but it also offers displaced Syrian physicians and health professionals the opportunity to enter the workforce in their field of expertise. Presently, there are 154 Migrant Health Centers offering services in 27 provinces and operating 527 migrant health units—all run in coordination with the Ministry of Health. In areas where the Syrian population exceeds 20,000, the Ministry of Health has also developed expanded versions of the Migrant Health Centers. These health facilities are better-equipped and offer secondary and tertiary health services in addition to primary care. The goal of these expanded centers is to provide better quality of care and to reduce the burden on state-run hospitals treating primarily Turkish citizens. Notably, data on the effectiveness of the Migrant Health Centers for chronic disease detection and treatment is limited [30]. Though Turkish law dictates the provision of health services for Syrian migrants, several regulations still exist. Syrians living in urban settings can only seek healthcare in the city or governorate in which they have registered. Regardless of their health condition, except emergency cases, Syrians must make an online appointment or go directly to a primary health provider to set up an appointment. Syrians under TPR are not allowed to approach secondary and tertiary care without a referral from a primary care provider. Each registered Syrian asylum seeker is provided with an identity card that includes a Foreign Identification Number (FIN) that is unique to the individual. Upon arriving to a primary health care center, a patient’s FIN is used to check them into MEDULA, the national health insurance information system in Turkey, and to ensure their legal status within Turkey. If confirmation of their identity and status is infeasible, no healthcare services are provided. If the healthcare institution in question does not provide the services requested, in most cases, the patient will be referred to another healthcare center with those services, even if it is outside the city or governorate of registration. Table 1 below reports data obtained from the Turkish Ministry of Health on health services provided to Syrian migrants under Temporary Protection as of 2018.

### 3.3. Barriers to Health Services

While Turkey has made great strides in providing primary care for its population of Syrian refugees through universal healthcare, the HTP, and the Family Medicine model, its healthcare system in its current state is not adequately equipped to address the chronic disease burden of this population [11]. Although Turkey has guaranteed individuals under TPR full access to health services, practical challenges and barriers to accessing healthcare among this vulnerable population still exist. While Syrian refugees consider primary health care one of their top health needs, only about a third believe they could access health services for chronic diseases, if needed [8]. Among those in need of chronic disease care, nearly half reported a barrier to receiving this care [8]. Based on interviews with stakeholders to the refugee crisis and our literature review, we identified five challenges faced by Syrian refugees in Turkey in accessing healthcare services: registration procedure regulations, navigation of a new health system, language, fear of adverse treatment, and cost.

#### 3.3.1. Registration Procedure Regulations

Updated registration procedures have continued to pose a barrier to Syrian refugees seeking medical attention. To access health services, Syrian migrants must be registered with an identity card number. Originally, migrants were given personal numbers beginning in 98. However, after the introduction of TPR in 2014, newly incoming migrants were given personal numbers starting in 99, and all individuals already having migrated to Turkey were asked to re-register to receive personal identity numbers beginning in 99. Due to this change, individuals who did not have a personal identity number beginning in 99 were not able to access health services. 

The electronic health system that Turkey utilizes only recognizes personal identity numbers beginning with 99, and as a result, Syrians who were unable to re-register or were unaware of such policy changes are unable to access healthcare services free of charge. Each governate within Turkey has its own registration protocol, and though re-registration is widely considered to be available year-round, multiple stakeholders have reported that registration is only open at certain points of the year. Registration entities make registration available to those seeking TPR based on the volume of refugees coming into a given governate during a period of time as a means to ensure that there are enough services and resources to keep up with the growing number of individuals eligible to access those services. In these cases, displaced migrants can go weeks and even months without access to any services, which can be detrimental to health outcomes and overall integration. At present, due to overcrowding and back-up in registration procedures, delays have ensued and thousands of individuals, depending on their original province of registration, have been left without documentation to confirm their status under TPR and their resulting access to health services. 

#### 3.3.2. Navigating the Turkish Healthcare System

The Syrian healthcare system is vastly different from that of Turkey, and as a result, Syrians struggle in navigating and understanding the Turkish health system. For instance, amidst the transformation in the Turkish health system, Syrian migrants have been reported to have struggled to understand the referral-based system in place. Additionally, Syrian refugees may not be well informed about what health services are available in Turkey and what services they are entitled to under TPR. It is important that host countries extending such provisions make the additional effort to keep these vulnerable populations as informed as possible or to create a centralized system that has the capacity to communicate system changes on a population level. 

#### 3.3.3. Language

Since the beginning of the influx of Syrian refugees in Turkey in 2011, language has been a major barrier in allowing Syrians to access a variety of services, including healthcare. Both in making appointments and attempting to seek medical attention, Syrian refugees struggle to understand Turkish. For instance, given that Syrians mostly encounter emotional conditions [32], the language barrier severely limits access to services in which the patient must express themselves and communicate clearly with the health professional involved. Other studies have identified language as a major barrier to care and have further highlighted both the challenges and complexities of health service provisions and the importance of addressing this barrier in order to provide high-quality, accessible care [33,34]. Despite recent advances to provide interpreters and Syrian professionals who speak Arabic in Migrant Health Centers [35], not all health facilities in Turkey have capable interpreters. If the recent reforms instituted by the Ministry of Health to provide greater language accessibility continue to expand across Turkey, the language barrier may be alleviated as Syrians would be able to access services in their native language. 

#### 3.3.4. Fear of Adverse Treatment

The integration of Syrians into Turkish society has precipitated anti-immigrant sentiments, further compounding refugees’ barriers to healthcare access [36]. In the 2015 Turkish Perceptions Survey, 84 percent of Turkish people reported that they were concerned about refugees coming from Syria, and 68 percent wanted Turkey to adopt more restrictive immigration policies [37]. An overwhelming 73 percent of respondents reported that existing Syrian refugees should be asked to return home. These negative perceptions may deter Syrians from seeking out health services out of fear of adverse treatment, which may also contribute to unequal access to health services and resources. Similarly, refugees may not register out of fear of prosecution by Turkish authorities. Refugees who have not registered do not have guaranteed access to health services [38]. 

#### 3.3.5. Cost 

Our literature review revealed that cost was the most commonly cited barrier to healthcare access for Syrian refugees, namely provider, transportation, and medication costs [39,40,41]. The high cost of prescription medications is of particular importance, as many chronic health conditions require long-term medication regimes [42]. Though prescription medications are covered by the Turkish health system for registered refugees with TPR, cost poses a barrier to refugees who are unregistered or who seek care at private clinics. Even for those under TPR, it has been consistently reported that delays in providing reimbursements by government entities to pharmacies and hospitals providing medications to displaced persons has resulted in these dispensers refusing to provide refugees with prescription medications. As such, there is an urgent need to identify sustainable, long-term solutions that will facilitate access to affordable health services and medications [39]. While investing in more initiatives for chronic disease prevention and treatment may be perceived as presenting a substantial financial burden on Turkey’s health system, this approach may actually be cost-saving by reducing future hospitalizations and more aggressive treatments associated with undiagnosed or poorly managed chronic diseases and their subsequent complications [38].

## 4. Discussion

Based on our review of the Turkish healthcare system, we discuss our findings and provide the following recommendations to improve health services and healthcare access, with a particular emphasis on prevention, detection, and the treatment of chronic diseases among a vulnerable Syrian refugee population. 

The first crucial step in ensuring Syrian refugees are able to engage with the Turkish healthcare system is to ensure timely and accessible registration within days of arrival. Increasing human resources and removing language barriers during the dissemination of information related to registration procedures and the registration process itself has the potential to facilitate equal access to primary health care services [43]. Ensuring equitable registration will also lessen the financial burden of care as primary care services are provided free of charge to registered refugees. Additionally, it is important to note that some refugees may be unaware of their chronic health conditions, necessitating the need for the host health system to ensure timely detection to facilitate treatment and prevent the development of complications [44].

Once Syrians are registered and able to receive primary care health services free of charge, they are still likely to encounter a number of barriers as previously discussed. These barriers to healthcare access are a primary concern for improving care among Syrian refugees with chronic health conditions. As such, efforts should be targeted to address these barriers to the greatest extent possible. For example, special attention should be given to continue to minimize language barriers to healthcare access. Removing barriers to access has been consistently identified as pertinent to improving health outcomes [45].

We also recommend paying particular attention to refugees of older age. Among the vulnerable Syrian refugee population, older refugees are often overlooked, and this demographic typically has higher rates of chronic illness as compared to other age groups [12,46]. Among Syrian refugees living in Turkey in particular, those aged 60–69 have the highest risk of chronic disease [47]. Although Syrians over the age of 60 constitute the lowest proportion of the Syrian refugee population living in Turkey, Turkey still has incentives to cater to the needs of this subgroup [48].

Given the limited research available to evaluate the provision of health services for chronic diseases among Syrian refugees living in Turkey, we also recommend strengthening the evidence base of the chronic disease burden and barriers to healthcare services for Syrian refugees in Turkey. Stronger evidence has the potential to drive healthcare policy and reform changes such that current gaps in care can be addressed and services can become more accessible. Syrian migrants in Turkey are vulnerable to a large array of chronic health conditions that require long-term access to healthcare in their new host communities. Despite the advances made by the Ministry of Health in recent years, more research is required to evaluate the effectiveness of Turkey’s health reforms for refugees on Syrian access to health services for chronic diseases. With a more thorough understanding of the current Turkish healthcare climate for refugees, efforts can be made to improve the quality of care and healthcare access for Syrian refugees in need of healthcare services for chronic disease prevention, detection, and treatment. 

Financing these recommendations, and any improvement to the health system for that matter, will add to the growing cost that the Turkish government has been harboring. Turkey has distinguished itself in its unique strategy and response to the influx of refugees and its ability to finance the cost of interventions largely from its government. In contrast to other countries facing massive influxes of refugees, such as Lebanon and Jordan, Turkey has not relied extensively on the support of humanitarian agencies. In fact, in Lebanon, Syrian refugees rely exclusively on humanitarian organizations for healthcare, with no national health insurance schemes to finance treatments costs, and in Jordan, since 2014, Syrian refugees have been required to pay a “non-insured Jordanian” rate, which is about 35–60% of what foreigners without insurance pay [49,50]. Having already spent $30 billion dollars to address the refugee crisis, Turkey must receive more support and must coordinate its interventions with humanitarian organizations to more adequately address the protracted, broad, and multi-faceted nature of this crisis. 

## 5. Conclusions

Despite the large number of Syrian refugees living in Turkey, little information is available in the existing literature to assess the chronic health challenges displaced Syrians face, as well as the challenges faced by the Turkish health system in the provision of healthcare for chronic diseases. In our study, we have evaluated the provision of chronic health services for Syrian refugees living in Turkey. Our study findings add to the scant literature on this important topic and provide actionable recommendations to the Turkish stakeholders engaged in providing healthcare to Syrian refugees with chronic diseases.

## Figures and Tables

**Table 1:** Total Data on Health Services Provided to Syrian migrants under Temporary Protection across Turkey (As of 30 April 2018) (Source: Turkish Ministry of Health).

Total Data on Health Services Provided to under the Temporary Protection across the Country		From 2011 to 2016 Total	2017	February	March	April	2018
			Total	2018	2018	2018	Total
Policlinic	Given by MoH Primary Health Services	5,742,528	3,005,287	318,568	385,486	330,146	1,354,398
	Given by MoH Secondary and Tertiary Health Services	16,997,636	8,291,891	787,153	861,431	810,493	3,242,680
	Given by University and Private Hospitals	328,338	136,127	20,483	26,389	23,488	91,214
	Total	23,068,502	11,433,305	1,126,204	1,273,306	1,164,127	4,688,292
Referral Data	From Primary Health Services to Public/University/Private Hospitals	918,964	N/A	9879	10,077	9683	29,639
	Total	918,964	N/A	9879	10,077	9683	29,639
Inpatient Data	Public Hospitals	1,018,271	357,834	33,341	37,263	32,561	138,749
	University/Private Hospitals	37,364	10,375	1409	1798	1523	5993
	Total	1,055,635	368,209	34,750	39,061	34,174	144,742
Surgery Data	Public Hospitals	854,318	311,164	27,902	36,778	30,221	120,862
	University/Private Hospitals	23,781	3703	442	629	518	1953
	Total	878,099	314,867	28,344	37,407	30,739	122,815
Delivery Data	Public Hospitals	195,421	100,671	8491	9062	8011	34,529
	University/Private Hospitals	4737	1639	192	231	204	764
	Total	200,158	102,310	8683	9293	8215	35,293
	Wounded Total	36,897	5425	1022	867	861	3302
